# The production of ginsenosides in hairy root cultures of American Ginseng, *Panax quinquefolium* L. and their antimicrobial activity

**DOI:** 10.1007/s11627-012-9469-5

**Published:** 2012-11-13

**Authors:** Ewa Kochan, Małgorzata Wasiela, Monika Sienkiewicz

**Affiliations:** 1 Pharmaceutical Biotechnology Department, Medical University of Lodz, ul Muszyńskiego 1, 90-151 Lodz, Poland; 2 Medical and Sanitary Microbiology Department, Medical University of Lodz, pl. Hallera 1, 90-647 Lodz, Poland

**Keywords:** Hairy root clones, Ginsenosides, Minimal inhibitory concentration

## Abstract

*Panax quinquefolium*, American ginseng, is valued for its triterpene saponins, known as ginsenosides. These constituents possess a number of pharmacological properties and hairy root cultures can synthesize similar saponins to those of field-cultivated roots. The antibacterial activity of extracts from three hairy root clones of *P. quinquefolium* L. was tested against a range of standard bacterial and yeast strains. The agar diffusion method was used to evaluate inhibition of microbial growth at various extract concentrations. Commercial antibiotics were used as positive reference standards to determine the sensitivity of the strains. Susceptibility testing to antibiotics was also tested using the disk diffusion method. The minimal inhibitory concentration values of the extracts, obtained by agar diffusion, ranged from 0.8 to 1.4 mg/ml. The results showed that extracts from hairy root cultures inhibited the growth of bacteria and yeast strains and suggest that they may be useful in the treatment of infections caused by pathogenic microorganisms.

## Introduction

**Figure 1.**
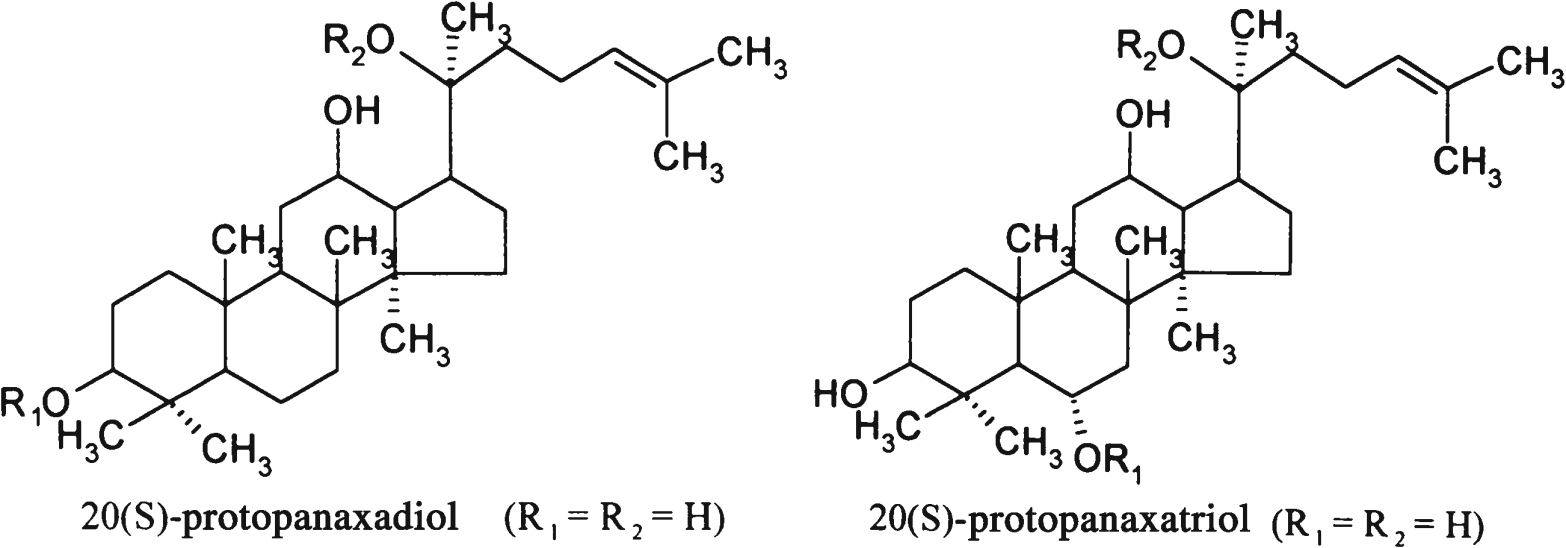
Chemical structure of protpanaxadiol and protopanaxartiol.

**Figure 2.**
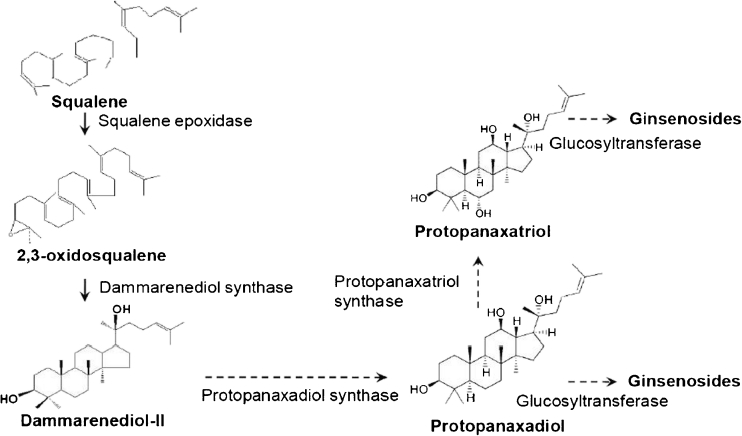
Biosynthetic pathway of ginsenosides from squalene in *P. ginseng*. Triterpene undergoes oxidation, glycosylation and is finally converted into triterpene saponins (ginsenosides), according to Kim et al (2009).

American ginseng (*Panax quinquefolium*) is an important medicinal plant belonging to the *Araliaceae* family. It is native to the hardwood-forested regions of the Eastern part of North America, but it has been successfully cultivated in New Zealand, China, Australia, Holland, France, and Poland (Kołodziej et al. 2006). However, ginseng is a slow-growing plant, difficult to cultivate in the field, and prone to disease. Saponins, a type of ginsenoside, the pharmacologically active ingredient, can be obtained only after 4–7 yr of cultivation. Therefore, hairy root *in vitro* cultures of *P. quinquefolium* provide an attractive alternative for obtaining the biologically active compounds. The two major groups of ginsenosides are Rb and Rg groups, derived from the 20(S) protopanaxadiol and 20(S) protopanaxartiol structures, respectively. The main compounds are the ginsenosides Rb1 (20(S) protopanaxadiol-3-[O-*β*-d-glucopyranosyl(l→2)-*β*-d-glucopyranoside]-20-O-*β*-d-glucopyranosyl(1→6)-*β*-d-glucopyranoside), Rb2 (20(S)-protopanaxadiol-3-[O-*β*-d-glucopyranosyl(l-2)-*β*-d-glucopyranoside]-20-[O-α-l-arabinopyranosyl(l→6)-*β*-d-glucopyranoside]), Rc (20(S)-protopanaxadiol-3-[O-*β*-d-glucopyranosyl(l→2)-*β*-d-glucopyranoside]-20-O-α-l-arabinofuranosyl (l→6)-*β*-d-glucopyranoside), and Rd (20(S)-protopanaxadiol-3-[O-*β*-d-glucopyranosyl(l→2)-*β*-d-glucopyranoside]-20-(O-*β*-d-glucopyranoside) from the Rb group, and Re (Re-20(S)-protopanaxatriol-6-[O-α-l-rhamnopyranosyl(l→2)-*β*-d-glucopyranoside]-20-O-*β*-d-glucopyranoside), Rg 1 (Rg1–20(S)-protopanaxatriol-6,20-di-O-*β*-d-glucoside) from the Rg group (Figs. 1 and 2; Table 1). Ginsenosides are responsible for most of the therapeutic action of ginseng (Yuan et al. 2010).

**Table 1.** Sugar molecules in the structures of ginsenosides examined in this study

Metabolite	R_1_	R_2_
20(S)-protopanaxadiol	H–	H–
Rb_1_	Glc–Glc–	Glc-Glc–
Rb_2_	Glc–Glc–	Glu–Ara
Rc	Glc–Glc–	Glu–Ara
Rd	Glu–Glu–	Glc–
20(S)-protopanaxatriol	H–	H–
Re	Glc–Rha	Glc–
Rg_1_	Glc–	Glc–

Experimental research has identified a range of pharmaceutical activities possessed by many secondary plant metabolites including polysaccharides, flavonoids, coumarins, glycosides, phenolic acids, saponins, and also essential oils. Many have strong antibacterial, antifungal, antiviral, anti-inflammatory, antioxidant, and also anticancerogenic properties (Sparg et al. 2004; Chanwitheesuk et al. 2005; Ahn et al. 2006; Sienkiewicz et al. 2011). Opportunistic infections caused by Gram-positive cocci belonging to *Staphylococcus* spp., *Enterococcus* spp., and Gram-negative bacteria, mainly of the *Klebsiella* spp., *Escherichia* spp., *Pseudomonas* spp., *Proteus* spp., *Enterobacter* spp., and *Serratia* spp. present rising problems (Wimmerstedt and Kahlmeler 2008; Foucault et al. 2009; Sharma et al. 2010). In addition, the broad and complex activity of plant metabolites, as well as their synergy of action, can make them a valued weapon against multidrug resistant bacterial strains (Tan and Vanitha 2004; Mahesh et al. 2008; Yang et al. 2012).

Currently, there is no literature concerning the antibacterial and antifungal activity of the extracts obtained from *P. quinquefolium* hairy root cultures. In this study, we investigated antimicrobial properties associated with ginseng hairy root extracts.

## Materials and Methods

### Hairy root cultures.

*P. quinquefolium* L. hairy root cultures were established from seedlings (obtained from field cultivation in the Agriculture University of Lublin, Lodz, Poland) after infection with an agropine-type strain of *Agrobacterium rhizogenes* ATCC 15834. After 6–8 wk, the roots emerged from the site of infection. When the roots were 1.5–2 cm long, they were individually excised and transferred into a hormone-free B-5 liquid medium with addition of 500 mg/l ampicillin. An aseptic culture of hairy roots was obtained, which grew rapidly on the same medium without antibiotic supplementation. Three clones, A, B, and G, were obtained and transformation was verified by PCR analysis (Kochan et al. 2012). Hairy root cultures were grown in 300 ml shaken Erlenmeyer flasks with 80 ml of hormone-free liquid B-5 medium (Gamborg et al. 1968), containing 30 g sucrose. The average inoculum size was about 300 mg fresh weight and 0.30 mg dry weight (dw). The cultures were maintained in the dark at 26°C on a rotary shaker (100 rpm) and subcultured every 28 d. Fresh roots, after drying on absorbent paper, were dried at room temperature and were processed for ginsenoside extraction and HPLC analysis as previously described (Kochan et al. 2008).

### Standard strains.

For antibacterial and antifungal activity testing, five standard strains of Gram-positive bacteria were used: *Staphylococcus aureus* ATCC 433000, *S. aureus* ATCC MR3, *Enterococcus faecalis* Van B ATCC 51299, *E. faecalis*, vancomicin-sensitive ATCC 29212, *Enterococcus faecium*, and vancomicin-sensitive ATCC 35667. In addition, four Gram-negative bacteria were used: *Escherichia coli* ATCC 35218, *E. coli* ATCC 25922, *Pseudomonas aeruginosa* ATCC 27853, and the yeast strain *Candida albicans* ATCC 10231. These standard bacterial and yeast strains used in the agar dilution method came from the collection of the Medical and Sanitary Microbiology Department, Medical University of Lodz, Poland.

### Drugs and bacteriological media.

Three antimicrobial agents were used: 5 μg/ml ciprofloxacin (Graso), a mixture of 20 μg/ml amoxicillin 10 μg/ml clavulanic acid (Graso); and 25 μg/ml fluconazole (Graso). The bacteriological media were Columbia Agar (bioMerieux), Mueller Hinton II Agar (EMAPOL), and Sabouroud Dextrose Agar (Graso).

### Suspensions of the tested bacterial strains.

The standard strains were cultivated in Columbia agar medium, incubated at 37°C for 48 h (bacteria) and in Sabouroud dextrose agar at 28°C for 48 h (yeast) in aerobic conditions. Bacterial and yeast suspensions were prepared with an optical density of 0.5 on the McFarland scale using a Bio Merieux densitometer.

### Susceptibility testing.

Susceptibility testing was carried out using the disk diffusion method (Jorgensen and Turnidge 2007). Cultures were incubated at 37°C for 16–18 h (bacterial strains) and 28°C for 24 h (yeast) in aerobic conditions. The results were interpreted according to Clinical and Laboratory Standard Institute guidelines (CLSI 2009).

### Antibacterial analysis using agar dilution method.

For antimicrobial testing, A1, A2, B1, B2, G1, G2 independent weighted samples respectively for clone A, B, and G were used. Each of the extracts from the hairy root clones was weighed, diluted in ethanol a concentration of 97% *w*/*v* of extracts and used as a stock solution. An appropriate amount of this solution was mixed with Columbia agar medium (bacteria) and Sabouroud dextrose agar (yeast) to obtain concentrations from 0.8 to 1.4 mg/ml, and these were dispensed into Petri dishes. An inoculum containing 1.5∙10^8^ CFU (0.1 ml) per spot was seeded either upon the surface of an agar plate with an extract from the hairy root clones (at various concentrations), or a plate with no extracts added (strains growth control).

The minimal inhibitory concentration (MIC) was determined after 24 h of incubation at 37°C for bacteria and at 28°C for yeast in aerobic conditions. The analyses of the antibacterial and antifungal activity of the extracts were performed independently three times. Control media containing only alcohol (at concentrations used in the dilutions of extracts) did not inhibit the growth of any of the bacterial or yeast strains.

## Results

### Characteristics of hairy root cultures.

Three independently generated hairy root *in vitro* cultures were established from sterile seedlings of *P. quinquefolium* that had be transformed with *A. rhizogenes.* For the 28-d culture period, the highest increase of dry biomass (above eightfold) was recorded for line A of *P. quinquefolium* hairy root culture, with a slightly lower increase for line G (sevenfold), and the lowest for line B (fivefold).

**Figure 3.**
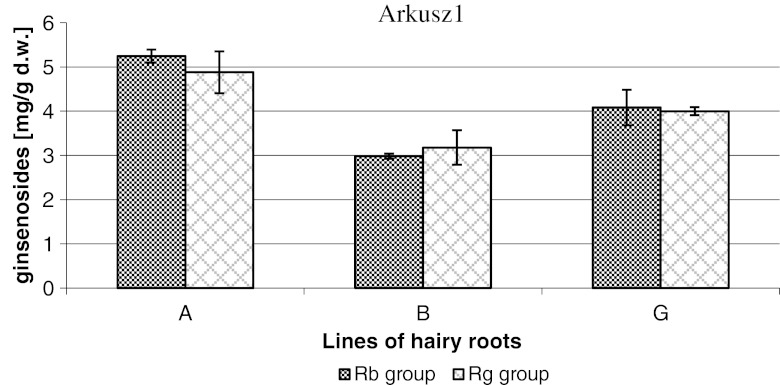
Ginsenoside contents of Rb group and Rg group in three lines of *P. quinquefolium* hairy roots.

The obtained root cultures synthesized six identifiable saponins: Rb1, Rb2, Rc, Rd, (derivatives of protopanaxadiol), and Rg1 and Re (derivatives of protopanaxatriol). Ginsenoside production (expressed in mg/g dw) after 28 d of culture is shown in Table 2 and Fig. 3.

**Table 2.** Ginsenoside content in three hairy root culture lines of *P. quinquefolium*

Lines of hairy roots	Ginsenoside [mg/g dw]						
	Rb1	Rb2	Rc	Rd	Rg1	Re	Total
A	3.934 ± 0.08	0.296 ± 0.019	0.9477 ± 0.11	0.066 ± 0.021	1.216 ± 0.14	3.662 ± 0.36	10.12 ± 0.52
B	1.933 ± 0.08	0.346 ± 0.038	0.6133 ± 0.03	0.037 ± 0.01	0.785 ± 0.07	2.384 ± 0.36	6.097 ± 0.44
G	3.169 ± 0.35	0.073 ± 0.015	0.7553 ± 0.07	0.085 ± 0.036	0.992 ± 0.07	3.006 ± 0.09	8.079 ± 0.34

Each value is a mean of six replicates ± SD

Clone A of the hairy roots achieved the highest total ginsenoside level (10 mg/g dw) followed by clones G and B. The content of individual metabolites in clones A, B, and G differed. The quantitative level of Rb1, Rb2, Rc, Rd, Re, and Rg1 saponins shows the pattern Rb1 > Re > Rg1 > Rc > Rb2 > Rd for clone A, with Rb1 being the most abundant and Rd the least. Clone G has similar pattern, but the content of ginsenoside Rd was slightly higher than Rb2. Clone B followed the same sequence as clone A, but the main ginsenoside was the metabolite Re (Table 2). Metabolite Rb was dominant among the protopanaxadiol derivatives, and metabolite Re was dominant among the protopanaxatriol derivatives in all examined lines. The sum of the main components (Rb1, Re) was more than 70% of total ginsenosides.

### Antimicrobial activity of *P. quinquefolium* extracts.

The MICs of the hairy root extracts (A_1_, A_2_, B_1_, B_2_, G_1_, and G_2_) against the tested microorganisms and susceptibility to standard strains of bacteria and yeast are shown in Table 3.

**Table 3.** MIC (milligrams per milliliter) for extracts of the hairy root of *P. quinquefolium*

Standard strain	MIC (mg/ml) of hairy root clone extract						Control of standard strains, susceptibility to antibiotics (mean dimeter zone of inhibiton (mm))			Ethanol
	A_1_	A_2_	B_1_	B_2_	G_1_	G_2_	CIP	AMC	FLU	
*S. aureus* ATCC 4330	0.9	0.9	1.2	1.2	1.1	1.2	24	ND	ND	NI
*S. aureus* ATCC MR3	0.8	0.9	1.4	1.4	1.3	1.4	22	ND	ND	NI
*E. faecalis* ATCC 29212 Van-	1.0	1.1	1.4	1.4	1.3	1.4	23	ND	ND	NI
*E. faecalis* ATCC 51299 VanB	1.1	1.3	1.4	1.4	1.4	1.4	23	ND	ND	NI
*E. faecium* ATCC 35667 Van-	1.0	1.2	1.4	1.4	1.2	1.3	22	ND	ND	NI
*E. coli* ATCC 35218	0.8	0.8	1.0	1.2	1.0	1.1	24	23	ND	NI
*E. coli* ATCC 25922	0.9	0.8	1.1	1.2	1.2	1.3	24	22	ND	NI
*P. aeruginosa* ATCC 27853	1.0	1.2	1.4	1.4	1.2	1.4	21	21	ND	NI
*C. albicans* ATCC 10231	1.0	1.0	1.4	1.3	1.2	1.2	ND	ND	28	NI

*CIP* ciprofloxacin (5 μg) (R ≤ 15, 16 ≤ I ≤ 20, S ≥ 21), *AMC* amoxicillin/clavulanic acid (20 μg/10 μg) (R ≤ 13, 14 ≤ I ≤ 17, S ≥ 18), *FLU* fluconazole (25 μg/ml) (R ≤ 14, 14 ≤ I ≤ 18, S > 18), *ND* not determined, *NI* not inhibited

The tested hairy root extracts were the most active against Gram-negative standard strains: *E. coli* ATCC 35218 and *E. coli* ATCC 25922. The MIC values were between 0.8 and 1.3 mg/ml. The extracts from clones A were found to be very effective against the standard *E. coli* strains (MIC 0.8–0.9 mg/ml). However, the MIC values of the growth inhibition factors in the extracts from all the clones were higher against *P. aeruginosa* (1.0–1.4 mg/ml).

*Enterococcus* sp. standard strains were the most resistant to tested hairy root extracts. The highest concentrations of extracts, between 1.1 and 1.3 mg/ml for A clones and 1.4 mg/ml for B and G hairy root clones, were effective against the vancomycin-resistant standard strain, *E. faecalis* Van B ATCC 51299. However, the MIC values for standard strain *S. aureus* ATCC 4330 were between 0.9 and 1.2 mg/ml and for *S. aureus* ATCC MR3 were between 0.8 and 1.4 mg/ml for the all clones. The MIC of the hairy root extracts against *C. albicans* ATCC 10231 was 1.0 mg/ml for the A clones, 1.2 mg/ml for the G clones and 1.3–1.4 mg/ml for the B clones. Overall, the strongest activity was observed by the A clones of the hairy root extracts. The control (ethanol) did not produce any inhibitory activity against the microorganisms.

## Discussion

The hairy roots of *P. quinquefolium* synthesized six types of saponins. The total ginsenoside level in lines A, G, and B of the hairy roots was found to be about 10, 8, and 6 mg/g dw, respectively. A lower level of total ginsenoside (2.58–5.44 mg/g dw) was reported by Mallol et al. (2001) in different phenotypes of hairy roots of *P. ginseng*. In the present study, ginsenosides Rb1 and Re were seen be the major component. Mathur et al. (2010) demonstrated that extracts of *P. quinquefolium* hairy roots also contained the highest level of metabolite Rb1 having protopanaxadiol as sapogenin and Re having protopanaxatriol as sapogenin after 4 wk of culture. The same metabolites, Rb1 and Re, dominated all 18 lines of *P. ginseng* hairy roots studied by Woo et al. (2004). Hairy roots of hybrid *P. ginseng* and *P. quinquefolium* growing on B-5 medium also synthesized six of the ginsenosides examined in this study, but only metabolite Rb1 significantly exceeded the level of the other saponins (Washida et al. 1998, 2004). The lowest level of saponin Rd, found in this study, was similar to that observed by Washida et al (1998, 2004) and Woo et al. (2004).

In our tests, standard strains of *S. aureus* (*n* = 2), *Enterococcus* spp. (*n* = 3), *E. coli* (*n* = 2), *P. aeruginosa* (*n* = 1), and *C. albicans* (*n* = 1) were sensitive to hairy root extracts at 0.8–1.4 mg/ml concentrations. The extracts demonstrated their highest activity against Gram-negative standard strains of bacteria. Similar results were obtained by De Villiers et al. (2010). In their investigations, methanolic extracts from the leaves of *Cussonia* sp. from the *Araliaceae* family were active against *P. aeruginosa* (MIC of 1.0–1.5 mg/mL) and *S. aureus* (MIC of 1.8 mg/ml).

In accordance with the literature, extracts from hairy root and untransformed roots of *Maytenus senegalensis* showed an inhibitory effect against the growth of *S. aureus* at concentrations in the range of 0.65–1.25 mg/ml. However, the root extracts of both untransformed and transformed root cultures were active against Gram-positive bacterial strains only (Jain et al. 2008). Using the agar well-diffusion method, *Glycyrrhiza glabra* root extracts were found to demonstrate significant antibacterial activity against two Gram-positive (*Bacillus subtilis* and *S. aureus*) and two Gram-negative (*E. coli and P. aeruginosa*) bacterial strains (Nitalikar et al. 2010).

Studies on the antimicrobial properties of the essential oils obtained from the cultured hairy roots of *Salvia miltiorrhiza* Bunge containing four diterpenoid tanshinones and three phenolic acids revealed that the compounds have very strong activity against Gram-positive and Gram-negative bacteria, and one fungal species (Zhao et al. 2011). Additionally, studies on the antimicrobial properties of Korean red ginseng confirmed that ginsenosides possess antibacterial activities toward pathogenic Gram-positive and Gram-negative bacteria. In our tests, the highest antimicrobial activity was demonstrated by clone A hairy root culture, which was specifically correlated with the high content of ginsenosides. The literature reports confirm not only the antimicrobial properties of ginsenosides, but also their synergy of action with commercial antibiotics, such as kanamycin and cefotaxime, on antibacterial activity against methicillin-resistant *S. aureus* strains (Sung and Lee 2008).

## Conclusions

1. Three studied lines of hairy root cultures of *P. quinquefolium* A, B, G synthesize six types of known ginsenosides. The highest level of saponins is reported for line A.
2. The tested extracts from hairy root clones inhibit the growth of standard bacteria and yeast strains. These have the potential to be used in combination with antibiotics in fighting infectious diseases.
